# Resolving Intra- and Inter-Molecular Structure with Non-Contact Atomic Force Microscopy

**DOI:** 10.3390/ijms160819936

**Published:** 2015-08-21

**Authors:** Samuel Paul Jarvis

**Affiliations:** School of Physics & Astronomy, University of Nottingham, Nottingham NG7 2RD, UK; E-Mail: Samuel.Jarvis@nottingham.ac.uk; Tel.: +44-115-846-8823

**Keywords:** atomic force microscopy, intramolecular, intermolecular, NC-AFM, supramolecular, hydrogen, bond

## Abstract

A major challenge in molecular investigations at surfaces has been to image individual molecules, and the assemblies they form, with single-bond resolution. Scanning probe microscopy, with its exceptionally high resolution, is ideally suited to this goal. With the introduction of methods exploiting molecularly-terminated tips, where the apex of the probe is, for example, terminated with a single CO, Xe or H_2_ molecule, scanning probe methods can now achieve higher resolution than ever before. In this review, some of the landmark results related to attaining intramolecular resolution with non-contact atomic force microscopy (NC-AFM) are summarised before focussing on recent reports probing molecular assemblies where apparent intermolecular features have been observed. Several groups have now highlighted the critical role that flexure in the tip-sample junction plays in producing the exceptionally sharp images of both intra- and apparent inter-molecular structure. In the latter case, the features have been identified as imaging artefacts, rather than real intermolecular bonds. This review discusses the potential for NC-AFM to provide exceptional resolution of supramolecular assemblies stabilised via a variety of intermolecular forces and highlights the potential challenges and pitfalls involved in interpreting bonding interactions.

## 1. Introduction

The ability to see a single atom, for many, was considered something of an impossible dream, with the smallest units of stable matter, that is, a unit still retaining identifiable chemical properties, remaining an abstract notion. Atoms were of course known to exist, but predominantly inferred from experimental measurements and only visualised via simulations or pictorial representations of their properties. That was until the invention of the field ion microscope (FIM) [1,2] and later the scanning tunnelling microscope (STM) [3,4,5], whose exceptional spatial resolution now lets us routinely visualise the atomic nature of materials in real space, allowing us to directly see single atoms. The development of the STM triggered a whole host of complementary methods, known under the umbrella term of scanning probe microscopy (SPM), which utilise almost every conceivable type of detectable tip-sample interaction. Arguably the most intuitive interaction of these is force, which has given us the ability to feel materials at the atomic and molecular level, much as we are familiar with interacting with everyday objects. This led to the development of one of the most challenging, yet exciting scanning probe methods for atomic scale interrogation: the non-contact atomic force microscope (NC-AFM) [6,7,8,9].

NC-AFM has provided ever more detailed insights into the atomic world and has been used to manipulate single atoms at room temperature [10,11], to yield chemical resolution [12] and to measure the force required to move atoms and molecules [13]. Arguably, however, one of NC-AFM’s greatest recent achievements has been to characterise single molecules with unprecedented detail, resolving their internal structure with atomic resolution. Although STM measurements have for many years revealed submolecular features, the STM is limited in that it provides information on the electronic structure of the molecule arising from its frontier molecular orbitals, which often bear little resemblance to the real atomic geometry.

Similar to the breakthrough that STM provided for imaging single atoms of crystal surfaces, NC-AFM (and in fact, the scanning tunnelling hydrogen microscopy (STHM) [14] variant of STM, as discussed later) can now directly provide beautiful real space images of the atomic structure of individual molecules, revealing a vivid appearance sharing an amazing similarity to school textbook ball-and-stick drawings. This unparalleled capability has led to an explosion of interest, fettered only by the difficulty in instrument operation and in achieving the highly-controlled environments required.

This review summarises many of the advances NC-AFM has made in characterising molecules with submolecular resolution. It begins with a summary of the techniques available to achieve submolecular resolution, collecting together many of the key studies now reporting images resolving features relating to the *intra*molecular structure of single molecules. The effect of flexure in the tip-sample junction is then discussed, which turns out to be essential for enhancing the appearance of the bond structure within molecules. The review then proceeds to discuss recent exciting work investigating assemblies of molecules stabilised via intermolecular forces, with a particular focus on hydrogen bonding, where the question is posed: can NC-AFM resolve and uniquely identify single intermolecular bonds? The many potential challenges in answering these questions are then discussed within the context of a larger number of supramolecular structures stabilised through a variety of intermolecular interactions.

## 2. Intra-Molecular Resolution-Resolving Internal Bond Structure with NC-AFM

In order to save both the reader (and the author) from a great deal of confusion, I wish to define at this point the terminology that shall be used throughout this review. From this point on, when referring to intra-molecular resolution, that is, resolving submolecular structure relating to the atoms and bonds within a single covalently bound molecule, I will instead refer to resolving the internal bond structure of the molecule. This distinction is made to avoid confusion when discussing effects arising from inter-molecular forces (that is, non-covalent interactions between molecules) and features that will often be discussed alongside internal molecular bond structure.

**Figure 1:**
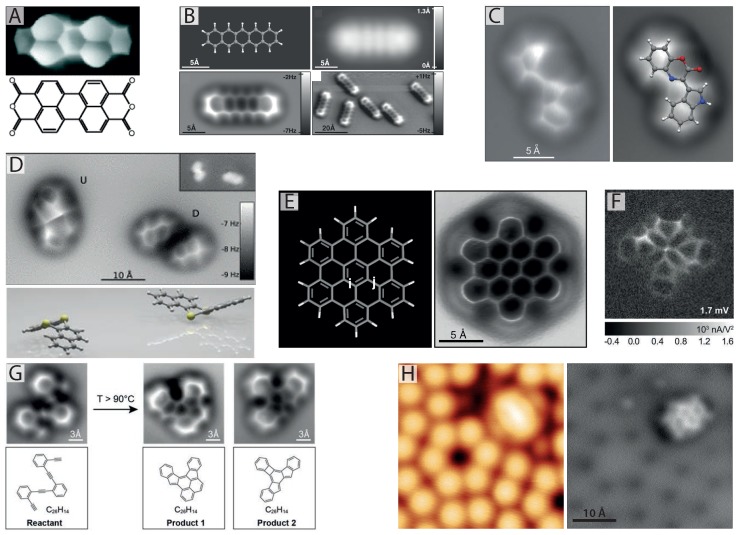
Imaging internal bond structure with CO-mediated non-contact atomic force microscopy (NC-AFM). (**A**) Internal bond structure of perylene-tetracarboxylic-dianhydride (PTCDA) resolved with scanning tunnelling hydrogen microscopy (STHM) using a trapped hydrogen molecule in the scanning tunnelling microscope (STM) tunnel junction [14,16]; (**B**) First images of internal bond structure resolved with NC-AFM achieved via pick-up of a single CO molecule onto the tip apex (from [24], reprinted with permission from AAAS); (**C**) NC-AFM images showing molecular structure identification of cephalandole A adsorbed on NaCl (2 ML)/Cu(111). Red and blue coloured atoms correspond to oxygen and nitrogen, respectively (reprinted by permission from Macmillan Publishers Ltd.: Nature Chemistry [27], copyright 2010); (**D**) NC-AFM reveals bistable configurations of dibenzo[a,h]thianthrene (DBTH) adopting a “butterfly” arrangement. Yellow coloured atoms correspond to sulphur (reprinted with permission from [28], copyright 2012 by the American Physical Society); (**E**) Pauling bond order discrimination in hexabenzocoronene with NC-AFM (from [29], reprinted with permission from AAAS); (**F**) Inelastic tunnelling spectroscopy (IETS) image revealing chemical bonds with a CO-terminated tip (from [30], reprinted with permission from AAAS); (**G**) NC-AFM images of the different steps of a chemical reaction with submolecular resolution (from [31], reprinted with permission from AAAS); (**H**) STM (left) and NC-AFM (right) imaging of the structure and adsorption site of naphthalene tetracarboxylic diimide (NTCDI) on the Si(111)-7×7 surface at 77 K [32,33].

The internal bond structure of a molecule (see reference [15] for an overview of submolecular resolution with various SPM techniques) was first observed by Temirov *et al.* [14] for perylene-tetracarboxylic-dianhydride (PTCDA) and tetracene, who introduced molecular hydrogen (H_2_) into an STM chamber during scanning. Due to the low temperature of the scan head (∼10 K), the H_2_ would spontaneously condense and trap itself within the tunnelling junction, significantly enhancing the resolution of the observed image (see Figure 1A). In this so-called scanning tunnelling hydrogen microscopy (STHM) mode of imaging, the position of the trapped H_2_ molecule is determined by the degree of Pauli repulsion felt within the small (<1 nm) tip-sample junction [16], causing the trapped molecule to effectively act as a transducer, modulating the STM signal via changes in the degree of Pauli repulsion. As will be described in more detail below, the effect of Pauli repulsion, which is largest when the tip is directly above the atoms and bonds of the molecule, is essential for achieving internal bond resolution. In addition to a H_2_ molecule, D_2_, CO, Xe and CH_4_ [17] have also all been shown to produce very similar results, demonstrating the general applicability of the STHM method.

Submolecular resolution in NC-AFM was first achieved using the qPlus [18] setup operated in the frequency modulation mode [19]. In the qPlus setup, the tuning fork is typically oscillated at its first eigenfrequency, usually at around 20–30 kHz. The relatively high stiffness of the tuning fork (nominally 1800 N·m^−1^, although measurements often vary [20,21]) enables sub-Angstrom oscillation amplitudes to be reached, which is often considered essential for atomic resolution imaging of molecules (although recent work now demonstrates that submolecular resolution is also achievable with cantilever AFM with large amplitudes up to ∼17 nm [22,23]).

In their seminal report, Gross *et al.* [24] successfully resolved the internal bond structure of pentacene using NC-AFM, shown in Figure 1B. Although somewhat similar STHM images had been published a year earlier, Gross *et al.* captured the clearest real space images of the internal bond structure of a molecule to date. Moreover, they were able to collect detailed quantitative information on the interaction responsible for imaging (as NC-AFM is sensitive to the tip-sample force gradient), making the images much simpler to interpret than the earlier STHM results. The fundamental mechanisms underlying contrast formation according to Gross *et al.* (at least to a first approximation) have an elegant simplicity, primarily relying on two prerequisites: (1) the tip must be chemically passivated, such that it weakly interacts with the surface-adsorbed molecule; and (2) the tip must be “sharp” such that its radius is sufficiently small to resolve atomic features. These two requirements enable the scanning probe to be placed extremely close to the surface-adsorbed molecule, such that a repulsive force is felt between the scanning probe and the molecule arising from Pauli repulsion [25,26]. Due to the strong localisation of the electronic density directly above the atomic positions of the molecule, the repulsion is strongest when the probe is positioned directly over the atoms and bonds. Therefore, a sufficiently sharp atomic probe can trace the corrugations of repulsion with atomic resolution, thus producing such exceptional images.

The first prerequisite in particular, that the tip must be chemically passivated, was essential for Gross *et al.*’s work (although it has since been observed that reactive tips can also achieve submolecular resolution when the molecule is strongly bound to the substrate [32,33,34].) Typically, either an etched W or cut PtIr wire is glued to the free tine of the tuning fork, thus acting as the tip. Without further tip functionalisation, these metallic tip structures make submolecular imaging on weakly binding substrates unfavourable, as the tip’s high reactivity usually induces lateral or vertical manipulation of the target molecule during imaging (long before the region where internal bond resolution can be achieved). To counter this problem, Gross *et al.* used a well-established technique from low temperature STM experiments [35,36,37] where a single small molecule is “picked up” from the surface and used to terminate the scanning probe apex.

Following a similar method to Bartels *et al.* [36,37], Gross *et al.* first ensured a sharp metallic tip by picking up single atoms of Au or Ag deposited onto a bilayer film of NaCl on Cu(111) (NaCl (2 ML):Cu(111)), achieved by approaching the scanning probe ∼4 Å towards the metal adatom (starting at a height defined by an STM tunnelling set point of *I* = 2 pA at 200 mV applied sample bias). Subsequently, the metal tip was then either terminated with a single CO molecule using a voltage pulse of 2.5 V, a Cl ion removed from the NaCl layer or one of the surface-adsorbed pentacene molecules. Out of these four tip terminations, only the CO- and Cl-terminated tips were able to resolve the surface-adsorbed pentacene with internal bond resolution, with the CO proving to be the most effective. In the case of an Ag/Au-terminated tip, Condition 1 was not met, and the target molecule was manipulated before the repulsive regime could be reached. For the pentacene-terminated tip, Condition 2 was not met, as the pentacene renders the probe sufficiently complex that a single sharp point is no longer present.

Interestingly, it was found that CO provided much greater apparent spatial resolution compared to Cl, which can be explained not only by the smaller spatial extent of the O atom’s electron density as compared to Cl, but also due to the effect of the flexibility of the CO molecule around the scanning probe, a topic discussed in much greater detail later in this review. In addition, Br and Xe tip terminations have also been tested [38], resulting in similar, albeit slightly poorer, improvements in resolution as found for CO-terminated tips. Due to the small size of the molecular termination, it is typically essential to perform experiments in a controlled environment at 5 K temperature, where diffusion can be avoided in order to maintain a stable molecular-terminated tip. Despite this, however, some studies have shown that submolecular resolution in NC-AFM can be achieved at temperatures as high as room temperature [23,39] and 77 K [22,32,40]. At 77 K , improved resolution was found to originate from spontaneous functionalisation of the scanning probe with much larger molecules, such as naphthalene tetracarboxylic diimide (NTCDI) [32,40] (where it was found that the C=O units of the NTCDI molecule behaved in a similar fashion to a single CO molecule) or the formation of TiO_2_ tip clusters [22]. It has also been shown that several other spontaneously occurring tip structures may give rise to submolecular resolution, particularly in semiconductor systems [32,41]. Other notable examples observing internal molecular bond structure with non-CO terminated tips were reported for decastarphene molecules on Cu(111) [42], 4-(4-(2,3,4,5,6-pentafluorophenylethynyl)-2,3,5,6- tetrafluorophenylethynyl)phenylethynylbenzene (FFPB) on Au(110) [43] and submolecular resolution observed on C_60_ molecules [44,45,46].

Since the initial report by Gross *et al.* [24] there has been an explosion of interest in CO-terminated probes applied to ever increasing numbers of molecular species, revealing exciting physics and chemistry at the submolecular scale. One of the most exciting prospects of CO-mediated NC-AFM is its ability to unambiguously reveal the unknown structure of molecular species, particularly in the case where other spectroscopic measurements, such as NMR, fail to identify a unique molecular structure. This was first shown by Gross *et al.,* who determined the structure of cephalandole A [27] adsorbed onto a thin film of NaCl, identifying a single compound out of a possible four (see Figure 1C). Later, the technique was also applied to much more complex molecules [47], breitfussin A and B, containing multiple chemical species, including I, Br, O and N. Despite the complexity of the molecular geometry, including deviations from a perfectly planar arrangement, NC-AFM images were able to resolve the detailed molecular architecture, greatly assisting in identifying a unique structural model and pointing towards the possibility of chemical identification. This highlights one of the major current challenges of the technique, as molecules deviating too far from a planar arrangement can be particularly challenging to image.

In addition to determining the internal structure of organic molecules, CO-mediated NC-AFM has shown excellent potential for determining conformational properties by directly imaging distortions of the molecular skeleton. For instance, the change in geometry of a PTCDA molecule during reversible covalent bond formation with a single gold atom [48] was observed. It was found that translation of the gold atom, initially located beside the molecule, to a position beneath it, caused a significant tilt in the molecule, clearly resolved in the NC-AFM image. Bistable configurations of dibenzo[a,h]thianthrene (DBTH) were also identified, and their adsorption site directly determined from simultaneous imaging of the molecule and surface [28,49]. Although STM measurements were able to differentiate between each conformer, only with the benefit of submolecular NC-AFM images were the exact adsorption geometries of each conformer identified (see Figure 1D). The adsorption geometries of several other molecules have also been identified [50,51], including members of the so-called olympicene family of benzopyrenes, which each showed variations in adsorption height and molecular tilt with respect to the surface plane when adsorbed on Cu(111).

Amazingly, beyond even imaging the internal bond structure of a molecule, CO-mediated NC-AFM provides important information regarding the *type* of atomic bond, specifically the bond order of individual carbon-carbon bonds within aromatic hydrocarbons. As demonstrated by Gross *et al.* in 2012 [29] variations in bond length corresponding to bond order were detected in NC-AFM images of C_60_ fullerenes and hexabenzocoronene (pictured in Figure 1E). The images revealed that carbon-carbon bonds with higher bond order consistently appeared shorter, and in some cases brighter (potentially arising from increased electronic density) than their lower bond order counterparts. As will be returned to later, the flexibility of the CO attached to the scanning probe is essential for providing such exquisite detail, as the flexible CO probe exaggerates the length of each bond, making the fractional distance between bonds of different order appear much larger in NC-AFM images than they really are.

There are several other important studies in the field worth highlighting before proceeding to discuss molecular assemblies. de Oteyza *et al.* have recently demonstrated NC-AFM’s capability to resolve the internal bond structure of single molecules at different steps during a chemical reaction (see Figure 1G and reference [31]), once again underlining NC-AFM’s ability to provide important insights into unknown chemical structures. It has now also been shown that internal bond resolution is achievable for a variety of substrates and tip terminations, such as NTCDI molecules adsorbed on the highly-reactive Si(111)-7×7 ( [32,33] and Figure 1H) and chemically-passivated Ag:Si(111)-($\sqrt{3}\times \sqrt{3}$) surfaces [40], both at 77 K, where spontaneous tip termination was observed to facilitate submolecular resolution. In addition, Moreno *et al.* [22] recently reported similar results with NC-AFM imaging of pentacene and C_60_ molecules on the (101) surface of anatase TiO_2_ also at 77 K, demonstrating the wide range of substrates on which internal bond resolution is now possible. In the same work, Moreno *et al.* also demonstrate submolecular imaging of non-planar molecules using a novel technique incorporating imaging in and out of feedback (with the out of feedback scan following the in-feedback profile at a reduced tip-sample separation). In very recent work, submolecular resolution in NC-AFM has now even been achieved under room temperature conditions [23,39]. Finally, in addition to NC-AFM, the use of CO-terminated tips is finding use in a number of complementary SPM techniques, such as Kelvin probe force microscopy (KPFM) and inelastic tunnelling spectroscopy (IETS). The combination of CO-mediated NC-AFM/STM and KPFM now allows unprecedented detail on the internal charge distribution within single molecules to be examined [43,52,53,54], and, with IETS [30], on the electronic and vibrational properties (see Figure 1F), opening the way for ever more detailed characterisation of a whole host of molecular properties in the coming years.

## 3. More than just an Image—The Effect of Tip Flexibility

Scanning probe microscopes are microscopes like no other; there are no lenses or mirrors, as light is not used to obtain an image. SPMs instead rely on sensing the interactions between an atomically-sharp cluster of atoms (*i.e.*, the scanning probe tip) and the surface material. SPM by its very definition is therefore invasive, and always affects the system under study in some way (although most of the time, we assume this is a minor effect). Indeed, the same interactions we exploit to acquire images are often used to manipulate those same individual atoms and molecules under study. Artefacts arising in STM and NC-AFM, therefore, unlike many optical techniques, cannot be shrugged off as unwanted effects, but are instead intrinsic to the technique and rooted in the underlying physical principles on which the microscopes operate. This provides an interesting challenge, as the simplicity and beauty of many SPM images often fails to convey the complexity of the microscope and methods required to obtain them. In other words, sometimes things are not quite as simple as just looking at an image.

**Figure 2:**
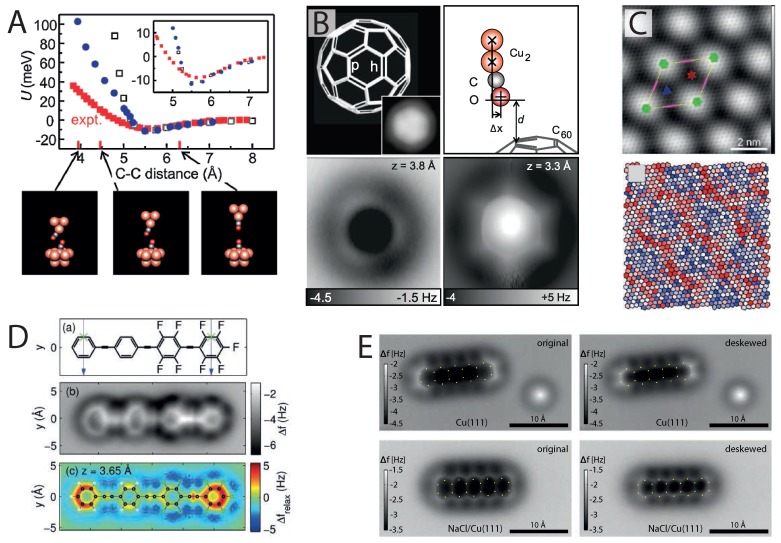
The effect of tip flexibility in high-resolution imaging. (**A**) CO bending observed in measurements of the CO–CO molecular pair potential (reprinted with permission from [55], copyright 2011 by the American Physical Society); (**B**) NC-AFM images showing bond length enhancement and sharpening due to flexibility at very small tip-sample distances (from [29], reprinted with permission from AAAS); (**C**) NC-AFM image showing variations in carbon ring size due to corrugation of Ir(111)-supported graphene (reprinted with permission from [56], copyright 2014 by the American Chemical Society); (**D**) Measurement and simulation of image distortions observed in NC-AFM images of 4-(4-(2,3,4,5,6-pentafluorophenylethynyl)-2,3,5,6-tetrafluorophe-nylethynyl) phenylethynylbenzene (FFPB) (reprinted with permission from [53], copyright 2014 by the American Chemical Society); (**E**) Image correction from lateral force analysis for pentacene imaged with NC-AFM on Cu(111) and NaCl (2 ML)/Cu(111) (reprinted with permission from [57], copyright 2014 by the American Physical Society).

This is particularly true in submolecular resolution imaging with NC-AFM. Even as early as the seminal 2009 paper by Gross *et al.* [24] it was noted that a Cl-terminated tip produced images where the carbon rings of pentacene appeared smaller in diameter compared with a CO-terminated tip, and did so without an asymmetry where the rings appeared elongated in one direction (particularly noticeable when imaged on NaCl (2 ML)/Cu(111)). This was followed by quantitative measurements of the molecular pair potential between two CO molecules by Sun *et al.* [55] who noted that the Cu-adsorbed CO molecules are far from an idealised rigid probe, and in reality show a great deal of flexibility, as shown in Figure 2A, making the point that “*... chemical repulsion between the CO molecules is relaxed at the expense of weaker bonding of the CO molecules to the Cu atoms of the tip and substrate, respectively*”.

As mentioned earlier, the flexibility of the CO probe was found to be essential for revealing the bond order of aromatic hydrocarbons. Detailed three-dimensional (3D) force maps collected above the hexagonal face of a surface-adsorbed C_60_ molecule [29] showed that at close tip-sample separations, the tilting of the CO is responsible for significantly amplifying the differences in apparent length of the p and h bonds, making the difference between them discernible within the lateral accuracy afforded by NC-AFM. Moreover, as shown in Figure 2B, tip-sample flexure also leads to a striking sharpening effect of the bonds, particularly at small tip-sample separations. Interestingly, similar to the noted difference between Cl- and CO-terminated tips, it was found that on the same dibenzo(cd,n)naphtho(3,2,1,8-pqra)perylene (DBNP) molecule, whilst a CO tip achieved exceptionally sharp resolution, once again enhancing the length of the aromatic bonds, a single-atom Xe tip showed no such distortion, providing an image much closer to the true atomic positions of the molecule [38]. This was attributed to the CO molecule’s greater ability to bend at the tip apex.

An elegant example illustrating the effect of flexure in the tip-sample junction was provided by Boneschanscher *et al.*, who investigated graphene on Ir(111) with CO-mediated NC-AFM [56]. Due to the lattice mismatch between graphene and the underlying Ir(111), the graphene buckles (following a moiré pattern), creating a smoothly-corrugated surface with a vertical distance ideally sized, such that internal bond resolution can be achieved simultaneously on both the top and hollow sites of the sheet. A single large NC-AFM image, taken at constant height, can therefore resolve a large number of equivalent carbon rings with a smoothly-varying change in tip-sample separation (and therefore, the degree of tip-sample repulsion) in an environment where edge effects and the asymmetry of the molecule are no longer a problem. Following this measurement, analysis of the area for each carbon ring revealed that hexagons at the moiré top sites were significantly larger than those in the hollow positions (see Figure 2C), showing deviations up to ±5% from the average value (after taking into account background forces), much larger than the accepted values of bond length variation. Once again, this was explained as due to variations in the degree of CO flexibility at different tip-sample separations. Moreover, the experimental data were supported by molecular mechanics simulations (modelled with a Lennard–Jones-type potential, see the next section) that modelled a flexible CO tip, fully reproducing the variation in apparent bond length, generating complete simulated images at relatively little computational cost.

An interesting result arising from the Lennard–Jones model was the observation of distorted carbon rings in simulations of pentacene [56], where the carbon rings appear elongated across the molecule’s short axis, taking on a similar appearance to the initial experimental observations on NaCl [24]. This effect was particularly noticeable for tips modelled with a smaller lateral spring constant describing the bending of the tip-terminating CO molecule (0.3 N·m^−1^), *i.e.*, for a given lateral force, the distortions of the flexible tip were larger compared to those using a greater value of stiffness. (This model will be revisited in the next section when discussing apparent bonds). A similar observation was also made by Moll *et al.* who examined image distortions in a partially-fluorinated hydrocarbon molecule FFPB [53], as shown in Figure 2D. In this case, using a density functional theory (DFT) description, it was found that the variation in distortion between the fluorinated and non-fluorinated carbon rings depended on both variations in the electronic density across the molecule and the flexibility of the tip.

In an elegant experiment showcasing the precision of 3D force-field acquisition with NC-AFM, Neu *et al.* [57] demonstrated that a “scaling constant”, unique for each CO tip, can be determined by assuming that the in-plane distortion scales linearly with the lateral forces acting on the CO. The lateral forces are determined by first integrating the measured frequency shift (Δf(x,y,z)) twice over *z*, obtaining U(x,y,z), before then taking the lateral gradient in *x* or *y* obtaining the lateral forces, $F_{x}$ and $F_{y}$ [13]. The NC-AFM images were then distortion corrected via the linear relation, producing the de-skewed images shown in Figure 2E. What is particularly striking is the correction observed for pentacene adsorbed on NaCl, where the lateral forces were found to be considerably larger. Importantly, based on repeated measurements, Neu *et al.* noted that there is no universal value for the optimal scaling constant, such that its value is unique for each individual tip apex, necessitating that it must be determined for each individual experiment. Although 3D measurements are certainly challenging, this procedure could in principle be applied to much larger molecules, where more complex distortions and lateral forces are present, therefore obtaining images with significantly reduced distortion (similar to Xe-terminated tips), whilst retaining the increased clarity and sharpness of NC-AFM images obtained with CO-terminated tips.

A major downside of tip flexibility, as discussed in detail in the next section, is the appearance of spurious features in the bond structure of molecules. In the earlier mentioned study by Gross *et al.* [27], investigating the unknown structure of cephalandole A, shown in Figure 1C, an apparent bond, similar in appearance to the C–C bonds, was observed between the deprotonated nitrogen and one of the nearby C–H units. The feature was very tentatively assigned to potential hydrogen bonding; however, it was noted that such a feature was not reproduced in the DFT simulations. Similarly, the conformational determination of DBTH [28,49] (shown in Figure 1D) shows a pronounced sharp feature connecting the two sulphur atoms of the molecule, despite no such bond being present. This is in addition to more subtle observations, such as the images of non-bonded Au-PTCDA clusters [48]. Despite NC-AFM images clearly resolving the adsorbed Au atom separated ∼3 Å from the C–H units of a PTCDA molecule, faint connecting features can be seen between the two that might mistakenly imply bond formation.

It is therefore abundantly clear that relaxations in the tip-sample junction, particularly for CO-terminated tips, are critical to understand the appearance of molecules imaged with submolecular resolution, even in the most simple of cases. Whilst tip-induced distortions can be cleverly exploited to image molecules with exceptional clarity and reveal information even down to the Pauling bond order, they can equally pose significant challenges with respect to image interpretation and in some cases make identifying the “true” structure particularly problematic, as we will see in the following section.

## 4. Resolving Inter-Molecular Bonds—Fact or Fiction?

With the difficulties associated with interpreting whether features arise from tip flexibility or real molecular bonds, it is clear that one of the greatest current challenges of CO-mediated NC-AFM is to attain exceptional resolution, down to the bond order of individual C–C bonds, whilst still maintaining correct information on the real molecular geometry. This raises an important question: to what extent can we trust the features present in submolecular NC-AFM images? That the observed length of a C–C bond can appear almost double the size of its real value, far beyond typical covalent bond lengths, suggests that in cases of complex molecules and molecular assemblies, significant care must be taken in image interpretation. Investigations with flexible tips are therefore ongoing, with the limitations of the technique far from clearly understood.

**Figure 3:**
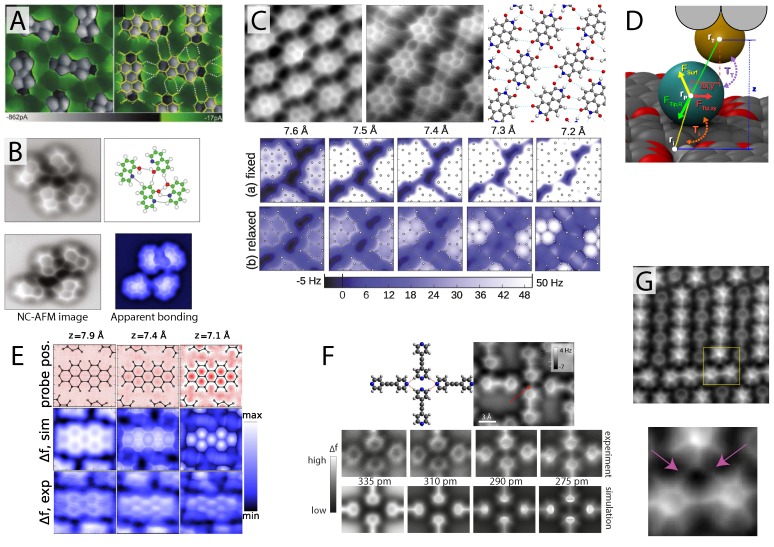
Intermolecular artefacts in hydrogen bonded assemblies. (**A**) First ever observation of apparent intermolecular bonds using scanning probe microscopy (SPM) observed via the STHM technique (reprinted with permission from [58], copyright 2010 by the American Chemical Society); (**B**) CO-mediated NC-AFM image of 8-hydroxyquinoline (8-hq) assembly exhibiting features in the locations of hydrogen bonding (from [61], reprinted with permission from AAAS) shown with simulated data (bottom right) [59]; (**C**) NC-AFM image revealing apparent intermolecular features in hydrogen bonded assemblies of NTCDI [40] also shown with simulated data confirming the apparent nature of the intermolecular features [59]; (**D**) Schematic of the flexible tip model used to simulate images of artificial intermolecular bonding [59]; (**E**) Experimental and simualted NC-AFM images of a PTCDA island shown with the simulated CO probe position [59]; (**F**) Experimental and simulated NC-AFM images of hydrogen bonded bis(para-pyridyl)acetylene (BPPA) molecules revealing apparent intermolecular bonds even where none are present (see red arrow) (reprinted with permission from [62], copyright 2014 by the American Physical Society); (**G**) NC-AFM image of apparent halogen bonding between the Fluorine atoms (see pink arrows in zoom of area marked with a yellow box) of three BPEPE-F18 (fluoro-substituted phenyleneethynylene) molecules (reprinted with permission from [63], copyright 2015 by the American Chemical Society). All figures from [59] reprinted with permission, copyright 2014 by the American Physical Society

The first SPM images of apparent intermolecular bonding were observed by Weiss *et al.* using the STHM technique on the herringbone phase of PTCDA on Au(111) [58] where both the internal bond structure of the molecule and sharp features extending across regions of intermolecular bonding were observed, as shown in Figure 3A. Later, it was shown that a variety of molecular terminations could achieve the same resolution [17], each resolving exceptionally-sharp features between the individual PTCDA molecules. Although for many years, the true origin of the observed contrast remained unclear, the molecular tip was assumed to mediate the interaction, somehow acting as a transducer, modulating the observed tunnel current signal via either Pauli repulsion or longer range electrostatic forces [16,17,58]. Similar to the results described below, through an elegant model incorporating flexible molecularly-terminated tips and a numerical model for the tunnelling process *through* the mediating molecule, it has been recently shown that the observed intermolecular features are a direct consequence of the flexible tip geometry [59,60].

CO-mediated NC-AFM images of molecules stabilised via intermolecular bonds were initially reported by Zhang and Chen *et al.* [61] who investigated assemblies of 8-hydroxyquinoline (8-hq) molecules stabilised via hydrogen bonding. In their report, a CO-terminated tip was argued to not only resolve the internal structure of 8-hq, but also to visualise features appearing between the molecules located in many of the expected locations for hydrogen bonding (see Figure 3B). The experimental measurements were presented alongside DFT calculations of the different observed structures, which, via an analysis of the charge density difference (CDD), confirmed the presence of hydrogen bonding, although its contribution to the total electron density (TED) was orders of magnitude smaller than that of the internal C–C bonds. (The TED has been used as a crude approximation for CO-mediated NC-AFM images, as to a first estimation, the regions of highest TED should correlate with the regions of highest Pauli repulsion sensed by the NC-AFM probe). Whether the intermolecular features were real, however, or simply an imaging artefact, remained unclear, as no account was taken of the tip-sample interactions in the molecule-only calculations presented (which, as described throughout the previous section, can have a profound effect on imaging).

Shortly after, we reported [40] very similar features observed for hydrogen-bonded assemblies of naphthalene tetracarboxylic diimide (NTCDI) molecules on the Ag:Si(111)-($\sqrt{3}\times \sqrt{3}$) surface, as shown in Figure 3C, where once again, exceptionally clear features were observed connecting the molecules together. In this report, the force field above the hydrogen bonded molecule was measured via collection of a high-density 3D grid of Δf(z) force-spectroscopy data. These results were compared with dispersion-corrected DFT calculations modelling the interaction of a number of tip terminations with the NTCDI island, which determined that an NTCDI-terminated tip, with its C=O group pointing towards the surface, was most likely responsible for observing the submolecular resolution. As an attempt to model the influence of the tip on the observed images, two-dimensional line profiles of *F(z)* curves were simulated across both the C–C and hydrogen bonds of the NTCDI island. Although this reproduced well the apparent height of the C–C and hydrogen bond features observed in the experimental images, the DFT calculations were unable to capture the striking sharp appearance of the apparent intermolecular features.

An important development in the interpretation of intermolecular features in CO-mediated NC-AFM came from Hapala *et al.* [59,60] and Hämäläinen *et al.* [62] who showed that the flexibility of the molecular tip, rather than the presence (or lack thereof) of an intermolecular bond, determines whether such interconnecting features are observed between hydrogen-bonded molecules, strongly suggesting that such features cannot be directly assigned to direct visualisation of intermolecular bonding. Using a similar model (available at the following link: [64]) to that described by Boneschanscher and Hämäläinen *et al.* [56], Hapala *et al.* modelled the molecularly-terminated NC-AFM probe as the outermost atom of a metal tip with a single probe particle at its apex. The probe particle was subject to three primary forces: (i) the tip-surface force, modelled as the sum of all Lennard–Jones forces acting between the probe and the molecular layer; (ii) a radial force connecting the probe particle to the tip base at a distance tunable to the particular molecular termination modelled; and (iii) a harmonic restoring force modelled with a lateral stiffness typically between 0.3 and 1.5 N·m^−1^ (see Figure 3D for a schematic). By tuning two primary parameters, namely the probe particle radius (to radii representing, for example, oxygen or xenon) and the lateral stiffness, Hapala *et al.* were able to reproduce almost all of the primary features observed in images of intermolecular and internal bond structure resolution images with NC-AFM and STHM [59], as well as IETS measurements [60]. The IETS simulations also suggested that submolecular resolution imaging may strongly depend on the electrostatic force, raising the intriguing possibility that such images could be used to obtain information on the surface electrostatic potential.

The most important aspect of the model is the complete absence of any electronic structure information, such as the electronic density associated with chemical bonding. Despite this, just a simple summation of Lennard–Jones forces, centred at the location of each constituent atom of the molecule, is enough to almost fully reproduce the experimental measurements. Figure 3E shows one such simulation for an island of PTCDA molecules where increased submolecular resolution is observed to directly correlate with the localisation of the probe particle position to regions of energy minima located off the molecular bonds, primarily inside the carbon rings and away from locations where adjacent molecules sit close to one another, such as regions of intermolecular bonding. This effect not only leads to a dramatic sharpening of the bonds observed in NC-AFM images, but due to the bending of the probe molecule, acts to normalise internal and intermolecular features, such that both are equally visible (compared to an inflexible tip structure, where internal features appear much brighter). The success of the model is demonstrated in numerous examples in the same paper, including the 8-hq and NTCDI systems shown in Figure 3B,C respectively, where experimental NC-AFM images were fully reproduced, despite the complete absence of intermolecular bonding between the molecules.

Despite such strong evidence, it is tempting to ask whether such distinctions between the origin for the features matter. After all, in the end, are not the features located at the hydrogen bonds anyway, whether they are directly responsible for the image contrast or not? This exceptionally important question was addressed by Hämäläinen *et al.* [62] in an elegant experiment examining bis(para-pyridyl)acetylene (BPPA) molecules, organised as tetramers in islands stabilised via hydrogen bonding. Due to clever experimental design, the molecules arrange end-on as tetramers, each forming a single hydrogen bond with its neighbour, but never the molecule directly opposite, as shown in Figure 3F. Importantly, not only do the opposing molecules face each other with two nitrogen atoms, unable to form a bond, but they do so at a separation comparable to the distance between the C–H and N atoms that can form a bond; therefore if a feature is observed in the N–N junction it can only be the result of apparent bonding due to probe relaxations, confirming the artificial nature of the observed features. This is confirmed in Figure 3F throughout a sequence of NC-AFM images taken at decreasing tip-sample separation, where not only are interconnecting features observed in the hydrogen bonding locations, but also in the non-bonded N–N junction. Moreover, using their own flexible tip model, the connecting features were fully reproduced in simulated images, with the apparent bond appearing with the same brightness as over the regions of hydrogen bonding.

It is then clear that in the case of predominantly electrostatic interactions, such as hydrogen bonding, the observation of apparent intermolecular features cannot be interpreted as the identification of real intermolecular bonds. Although, in exceptionally simple systems, a majority of features may well indeed correlate with real hydrogen bonds, with no prior knowledge with which to compare, CO-mediated NC-AFM simply cannot be used to identify the existence or not of hydrogen bonding. Indeed, very recent results on alternative systems now almost completely rule out that such features can be ascribed to intermolecular bonding. For example, measurements performed on fluoro-substituted phenyleneethynylene (bis(2,3,5,6- tetrafluoro-4-(2,3,4,5,6-pentafluorophenylethynyl)phenyl)-ethyne (BPEPE-F18) molecule [63] show that apparent bonds can be observed between C–F units of the molecule, as shown in Figure 3G. BPEPE-F18 interacts via halogen bonding, a purely electrostatic interaction with no accumulation of electron density between the molecules, therefore ruling out the possibility that NC-AFM directly images the bond itself. Additionally, unpublished results from our group [65] involving an examination of islands of close-packed C_60_ fullerene molecules also exhibit sharp, directional, interconnecting features between molecules, despite the purely van der Waals nature of the bonding interaction, the large separation between molecules, and the highly non-planar geometry. It therefore appears that despite a huge amount of excitement surrounding early results on hydrogen-bonded molecules, it is unfortunately the case that hydrogen bonds are neither directly imaged, nor can they be indirectly implied from the observation of apparent interconnecting features.

## 5. Prospects of NC-AFM in Supramolecular Studies

**Figure 4:**
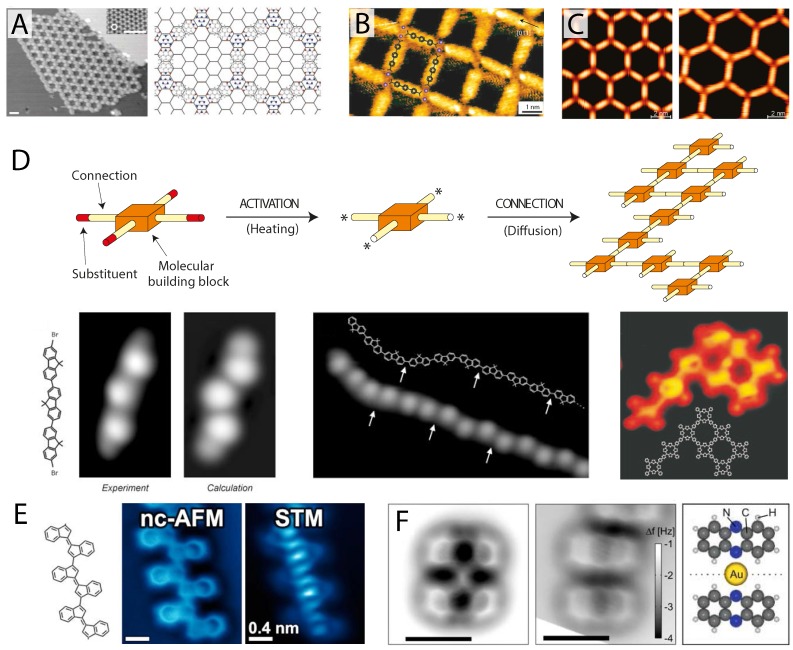
Potential for submolecular imaging of supramolecular systems. (**A**) STM image of a complex porous hydrogen bonded network comprising a mixed phase of perylene tetra-carboxylic di-imide (PTCDI) and melamine (reprinted by permission from Macmillan Publishers Ltd.: Nature [66], copyright 2003); (**B**) Nanocavities formed on Cu(100) by 4,1^′^,4^′^,1^″^-terphenyl-1,4^″^-dicarboxylic acid (TDA) molecules metal-coordinated with Fe atoms imaged in STM (reprinted by permission from Macmillan Publishers Ltd.: Nature Materials [88], copyright 2004); (**C**) Porous metal-coordinated networks of tunable size formed by NC-Ph*_n_*-CN molecules coordinated with Co atoms on Ag(111) imaged with STM (reprinted with permission from [89], copyright 2007 by the American Chemical Society); (**D**) Schematic and STM images of covalently-polymerised molecular assemblies of tetraphenyl porphyrin 2D networks (**left**) (reprinted by permission from Macmillan Publishers Ltd.: Nature Nanotechnology [90], copyright 2007) and dibromoterfluorene (DBTF) molecular wires (**right**) (from [91], reprinted with permission from AAAS); (**E**) CO-mediated NC-AFM (**left**) and STM (**right**) images of a covalently-linked oligomer following on-surface cyclisation on Au(111) (reprinted with permission from [92], copyright 2014 by the American Chemical Society); (**F**) CO-mediated NC-AFM image of a bonded (**left**) and non-bonded (**right**) phenazine-gold complex (schematic shows bonded geometry) (reprinted with permission from [93], copyright 2013 by the American Chemical Society).

There are a wide variety of intermolecular interactions available to stabilise 2D supramolecular networks. Even, for the moment, remaining within the confines of hydrogen bonding interactions, many complex structures can be formed exploiting a variety of molecules and hydrogen bond donors and acceptors. In the pioneering paper by Theobald *et al.* [66], for instance, a mixed phase of PTCDI and melamine was found to produce large porous networks of well-defined size, capable of templating the subsequent growth of C_60_ fullerenes, as shown in Figure 4A. In this case, the molecules arranged in a well-defined manner, maximising the number of hydrogen bonds in a way that can be easily understood. In addition, a recent AFM investigation has shown that such structures are stable across a range of insulating materials, even under ambient conditions [67]. In other systems with more complex arrangements [68,69,70] and varying molecular species [71,72,73,74], however, the location and number of hydrogen bonds formed is not always trivial to answer, often requiring simulation input. The prospect of a technique capable of single bond resolution is therefore extremely attractive, provided, of course, that the necessary care is taken in the image analysis so as not to mistakenly assign artificial interconnecting features as real bonds.

Prior to the advent of CO-mediated submolecular imaging, supramolecular systems were, and continue to be, studied in great detail with NC-AFM, primarily on bulk insulating substrates otherwise inaccessible by other techniques. These include, as reviewed in detail elsewhere [75,76], investigations spanning prototypical molecules, such as C_60_ [77,78] and PTCDA [79,80,81] to a variety of small molecules, many of which are capable of forming hydrogen bonded networks [82,83,84,85,86,87]. Central to these studies is the use of cantilever NC-AFM operated in feedback at room temperature with single molecule resolution. There is therefore significant potential for submolecular NC-AFM to complement these investigations and reveal even greater detail on their interactions.

There is of course much more to 2D supramolecular chemistry than hydrogen bonding, as discussed in many excellent review articles [94,95,96,97,98]. Molecular networks stabilised through metal coordination are a particularly interesting case as the strong coordination bonding interaction allows a variety of 2D molecular networks with tunable properties to be grown, such as networks with functional cavities [88] and tunable pore size [89] as shown in Figure 4B,C. The benefit of investigating such networks using CO-mediated NC-AFM lies in the fact that a sizeable coordinating metal atom must be present to mediate the bonding, providing a clear feature that could potentially be observed in the NC-AFM image. Moreover, metal coordination bonds tend to be longer than other forms of bonding, as there are typically two bonds formed with the coordinating atom, requiring that the molecules should be reasonably well separated. Apparent bonding due to tip flexibility and the close proximity of molecules should therefore be reduced, if not completely avoided. Although very few studies are currently available, Albrecht *et al.* [93] have examined co-adsorption of a phenazine and single gold adatoms on a thin film of NaCl on Cu(111) before and after formation of a metal-coordinated complex, as shown in Figure 4F. The phenazine-gold-phenazine complex is shown in the left panel of Figure 4F, with a non-bonded pair in the centre for comparison. Clearly, in the case of the metal complex, a distinct additional feature joins the two molecules together at their centre, as shown in the cartoon in the right panel, inducing a slight distortion to the molecule, causing the centre rings to appear darker (closer to the surface) than for the unbonded molecule.

As noted above, metal coordination networks benefit NC-AFM imaging, as the organometallic bond lengths are reasonably well defined and usually longer than that of the close proximity of features required to produce the apparent hydrogen bonds observed in the previous section. In some systems, it may even be possible to identify the position of the coordinating ion itself, which may appear much larger than a typical hydrocarbon atom (similar to a C≡C bond appearing significantly brighter and wider than a C–C bond, as seen in Figure 1G) offering much greater precision in characterising metal-coordinated structures than currently available via STM. On the other hand, metal-coordinating ions could be rather difficult to detect, due to their preference to sit closer towards the metal substrate than the molecules they bind to (and therefore, potentially obscured from the scanning probe, which typically operates at a constant height to obtain submolecular resolution). In these cases, novel protocols, such as the multi-pass lift-off technique proposed by Moreno *et al.* [22] or high-density 3D force field measurements, may become invaluable.

A final class of supramolecular structure—although, perhaps, use of the term supramolecular is no longer quite appropriate at this point—is the formation of large covalently-bound molecular networks. As originally shown by Grill *et al.* [90,91], single molecule precursor units, such as tetraphenyl porphyrin molecules, can be partially substituted with halogen atoms at their periphery. Following thermal activation on a catalytically-active surface such as gold or copper, the carbon-halogen bond is broken and the free radical molecules diffuse and bind together into 2D networks or 1D wires, depending on their design, stabilised via covalent C–C bonds (in effect, resulting in a single large molecule), as shown in Figure 4D. Examination of such covalently-bound structures with NC-AFM is now a particularly active area of research, with just a few examples currently published. In Figure 4E one such example is shown for Oligo-acetylene derivatives obtained following on-surface radical cyclisation [92]. Compared to hydrogen-bonded and metal-coordinated structures, the linking carbon bonds are not only exceptionally clear, but also appear with the same brightness as the linked molecules, which themselves exhibit no distortion from their isolated form. To an extent this should of course be expected, as the tip is once again imaging single C–C bonds, rather than intermolecular features. Although it is perhaps too early to tell, it may therefore be possible for NC-AFM to clearly distinguish between completely covalently-linked molecules and those simply still linked by precursor metalorganic bonds or exhibiting apparent bonding due to their close proximity, particularly in the case of less flexible tip terminations, such as Xe.

## 6. Conclusions and Perspectives

In the few short years since its first demonstration by Gross *et al.* in 2009, submolecular imaging with CO-mediated NC-AFM has experienced a huge amount of growth with broad application across a wide range of molecular systems. As the number of methods, substrate materials and molecular tip terminations available to achieve submolecular resolution increases, and ever more research groups overcome the technical difficulties associated with its implementation, adoption of the technique is reaching a tipping point, where we are now seeing the beginnings of a huge expansion of submolecular NC-AFM that will provide unprecedented insights into a vast array of molecular and supramolecular systems.

Many of the results reviewed here highlight the important interplay between the choice of molecular tip termination and its flexibility in determining the real atomic structure of molecules and their self-assembled structures. Whilst in many cases, the flexibility of the molecular tip can be cleverly exploited in order to reveal exceptionally-sharp resolution corresponding to internal bond structure, in some cases, even revealing the Pauling order of C–C bonds, it can equally induce distortions in the NC-AFM image causing significant deviations from the true atomic structure of the molecule. This is an important ongoing area of investigation, with techniques now being developed to apply image corrections based on lateral force measurement and background subtraction, but also alternative tip terminations that significantly reduce image distortions whilst still providing submolecular resolution (e.g., Xe and Br tip terminations). One route towards reducing tip flexibility may be not only to investigate more rigid or strongly tip-adsorbing probe molecule species, but also the preparation of more reactive metal tips [99], via controlled pick up of single metal atoms, capable of more stable, and potentially more directional bonding with a tip-adsorbed probe molecule. Additionally, recent observations of submolecular imaging on semiconducting and ionic substrates, such as silicon and TiO_2_, suggest that such surfaces may prove to be conducive for preparing rigid crystalline tip terminations. Often terminated with single hydrogen and oxygen atoms [22,100,101,102] such tip structures may dramatically reduce the effect of tip flexibility.

Understanding the nature of the tip-sample interaction and the best methods to reduce tip-induced distortions will be exceptionally important for future studies of supramolecular assemblies and on-surface chemistry [103]. Although in many respects, it is still early days, CO-mediated NC-AFM, operated and interpreted with care, may have the capability to offer unparalleled insight into supramolecular structures stabilised through a variety of intermolecular forces. As described in Section 4, there are already many studies reporting the exquisite detail achievable on hydrogen bonded molecular systems. Although NC-AFM cannot be said to directly image the hydrogen bonds themselves, or indeed accurately assign either their presence or absence, interpretation of the internal bond structure makes assignment of the orientation, separation and exact location of each individual molecule possible with single atom precision. This in itself may become invaluable in interpreting more complex supramolecular structures in the future. Over the coming years, the potential for NC-AFM to investigate various other supramolecular systems, such as those stabilised through metal-coordination and covalent bonds, will also be realised, opening the way for characterisation of molecular structures with unprecedented detail, pointing the way to an exciting future for NC-AFM in supramolecular chemistry.

## References

[1] Müller E.W., Bahadur K. (1956). Field ionization of gases at a metal surface and the resolution of the field Ion microscope. Phys. Rev..

[2] Müller E.W. (1965). Field ion microscopy. Science.

[3] Binnig G., Rohrer H., Gerber C., Weibel E. (1982). Surface studies by scanning tunneling microscopy. Phys. Rev. Lett..

[4] Stroscio J.A., Kaiser W.J. (1993). Scanning Tunneling Microscopy.

[5] Wiesendanger R. (1994). Scanning Probe Microscopy and Spectroscopy: Methods And Applications.

[6] Giessibl F.J. (2003). Advances in atomic force microscopy. Rev. Mod. Phys..

[7] Morita S., Wiesendanger R., Meyer E. (2002). Noncontact Atomic Force Microscopy, Vol. 1.

[8] Morita S., Giessibl F.J., Wiesendanger R. (2009). Noncontact Atomic Force Microscopy, Vol. 2.

[9] Morita S., Giessibl F.J., Meyer E., Wiesendanger R. (2015). Noncontact Atomic Force Microscopy, Vol. 3.

[10] Sugimoto Y., Abe M., Hirayama S., Oyabu N., Custance O., Morita S. (2005). Atom inlays performed at room temperature using atomic force microscopy. Nat. Mater..

[11] Sugimoto Y., Pou P., Custance O., Jelinek P., Abe M., Perez R., Morita S. (2008). Complex patterning by vertical interchange atom manipulation using atomic force microscopy. Science.

[12] Sugimoto Y., Pou P., Abe M., Jelinek P., Perez R., Morita S., Custance O. (2007). Chemical identification of individual surface atoms by atomic force microscopy. Nature.

[13] Ternes M., Lutz C.P., Hirjibehedin C.F., Giessibl F.J., Heinrich A.J. (2008). The force needed to move an atom on a surface. Science.

[14] Temirov R., Soubatch S., Neucheva O., Lassise A.C., Tautz F.S. (2008). A novel method achieving ultra-high geometrical resolution in scanning tunnelling microscopy. New J. Phys..

[15] Gross L. (2011). Recent advances in submolecular resolution with scanning probe microscopy. Nat. Chem..

[16] Weiss C., Wagner C., Kleimann C., Rohlfing M., Tautz F.S., Temirov R. (2010). Imaging Pauli repulsion in scanning tunneling microscopy. Phys. Rev. Lett..

[17] Kichin G., Weiss C., Wagner C., Tautz F.S., Temirov R. (2011). Single molecule and single atom sensors for atomic resolution imaging of chemically complex surfaces. J. Am. Chem. Soc..

[18] Giessibl F.J. (1998). High-speed force sensor for force microscopy and profilometry utilizing a quartz tuning fork. Appl. Phys. Lett..

[19] Albrecht T.R., Grütter P., Horne D., Rugar D. (1991). Frequency modulation detection using high-Q cantilevers for enhanced force microscope sensitivity. J. Appl. Phys..

[20] Sweetman A., Jarvis S., Danza R., Bamidele J., Gangopadhyay S., Shaw G.A., Kantorovich L., Moriarty P. (2011). Toggling bistable atoms via mechanical switching of bond angle. Phys. Rev. Lett..

[21] Shaw G.A., Pratt J., Jabbour Z. (2011). Small mass measurements for tuning fork-based atomic force microscope cantilever spring constant calibration. Conf. Proc. Soc. Exp. Mech. Ser..

[22] Moreno C., Stetsovych O., Shimizu T.K., Custance O. (2015). Imaging three-dimensional surface objects with submolecular resolution by atomic force microscopy. Nano Lett..

[23] Iwata K., Yamazaki S., Mutombo P., Hapala P., Ondráček M., Jelínek P., Sugimoto Y. (2015). Chemical structure imaging of a single molecule by atomic force microscopy at room temperature. Nat. Commun..

[24] Gross L., Mohn F., Moll N., Liljeroth P., Meyer G. (2009). The chemical structure of a molecule resolved by atomic force microscopy. Science.

[25] Moll N., Gross L., Mohn F., Curioni A., Meyer G. (2010). The mechanisms underlying the enhanced resolution of atomic force microscopy with functionalized tips. New J. Phys..

[26] Moll N., Gross L., Mohn F., Curioni A., Meyer G. (2012). A simple model of molecular imaging with noncontact atomic force microscopy. New J. Phys..

[27] Gross L., Mohn F., Moll N., Meyer G., Ebel R., Abdel-Mageed W.M., Jaspars M. (2010). Organic structure determination using atomic-resolution scanning probe microscopy. Nat. Chem..

[28] Pavliček N., Fleury B., Neu M., Niedenführ J., Herranz-Lancho C., Ruben M., Repp J. (2012). Atomic force microscopy reveals bistable configurations of dibenzo[a,h]thianthrene and their interconversion pathway. Phys. Rev. Lett..

[29] Gross L., Mohn F., Moll N., Schuler B., Criado A., Guitián E., Peña D., Gourdon A., Meyer G. (2012). Bond-order discrimination by atomic force microscopy. Science.

[30] Chiang C.I., Xu C., Han Z., Ho W. (2014). Real-space imaging of molecular structure and chemical bonding by single-molecule inelastic tunneling probe. Science.

[31] De Oteyza D.G., Gorman P., Chen Y.C., Wickenburg S., Riss A., Mowbray D.J., Etkin G., Pedramrazi Z., Tsai H.Z., Rubio A. (2013). Direct imaging of covalent bond structure in single-molecule chemical reactions. Science.

[32] Sweetman A.M., Jarvis S.P., Rahe P., Champness N.R., Kantorovich L.N., Moriarty P.J. (2014). Intramolecular bonds resolved on a semiconductor surface. Phys. Rev. B.

[33] Jarvis S.P., Sweetman A.M., Lekkas I., Champness N.R., Kantorovich L., Moriarty P. (2014). Simulated structure and imaging of NTCDI on Si(111)-7×7 : A combined STM, NC-AFM and DFT study. J. Phys. Condens. Matter.

[34] Boneschanscher M.P., van der Lit J., Sun Z., Swart I., Liljeroth P., Vanmaekelbergh D. (2012). Quantitative atomic resolution force imaging on epitaxial graphene with reactive and nonreactive AFM probes. ACS Nano.

[35] Eigler D.M., Lutz C.P., Rudge W.E. (1991). An atomic switch realized with the scanning tunnelling microscope. Nature.

[36] Bartels L., Meyer G., Rieder K.H. (1997). Controlled vertical manipulation of single CO molecules with the scanning tunneling microscope: A route to chemical contrast. Appl. Phys. Lett..

[37] Bartels L., Meyer G., Rieder K.H., Velic D., Knoesel E., Hotzel A., Wolf M., Ertl G. (1998). Dynamics of electron-induced manipulation of individual CO molecules on Cu(111). Phys. Rev. Lett..

[38] Mohn F., Schuler B., Gross L., Meyer G. (2013). Different tips for high-resolution atomic force microscopy and scanning tunneling microscopy of single molecules. Appl. Phys. Lett..

[39] Huber F., Matencio S., Weymouth A.J., Ocal C., Barrena E., J G.F. (2015). Intramolecular force contrast and dynamic current-distance measurements at room temperature. Phys. Rev. Lett..

[40] Sweetman A.M., Jarvis S.P., Sang H., Lekkas I., Rahe P., Wang Y., Wang J., Champness N.R., Kantorovich L., Moriarty P. (2014). Mapping the force field of a hydrogen-bonded assembly. Nat. Commun..

[41] Sang H., Jarvis S.P., Zhou Z., Sharp P., Moriarty P., Wang J., Wang Y., Kantorovich L. (2014). Identifying tips for intramolecular NC-AFM imaging via in situ fingerprinting. Sci. Rep..

[42] Guillermet O., Gauthier S., Joachim C., de Mendoza P., Lauterbach T., Echavarren A. (2011). STM and AFM high resolution intramolecular imaging of a single decastarphene molecule. Chem. Phys. Lett..

[43] Kawai S., Sadeghi A., Feng X., Lifen P., Pawlak R., Glatzel T., Willand A., Orita A., Otera J., Goedecker S. (2013). Obtaining detailed structural information about supramolecular systems on surfaces by combining high-resolution force microscopy with ab initio calculations. ACS Nano.

[44] Pawlak R., Kawai S., Fremy S., Glatzel T., Meyer E. (2011). Atomic-scale mechanical properties of orientated C60 molecules revealed by noncontact atomic force microscopy. ACS Nano.

[45] Pawlak R., Kawai S., Fremy S., Glatzel T., Meyer E. (2012). High-resolution imaging of C_60_ molecules using tuning-fork-based non-contact atomic force microscopy. J. Phys. Condens. Matter.

[46] Chiutu C., Sweetman A.M., Lakin A.J., Stannard A., Jarvis S., Kantorovich L., Dunn J.L., Moriarty P. (2012). Precise orientation of a single C_60_ molecule on the tip of a scanning probe microscope. Phys. Rev. Lett..

[47] Hanssen K.O., Schuler B., Williams A.J., Demissie T.B., Hansen E., Andersen J.H., Svenson J., Blinov K., Repisky M., Mohn F. (2012). A combined atomic force microscopy and computational approach for the structural elucidation of breitfussin A and B: Highly modified halogenated dipeptides from Thuiaria breitfussi. Angew. Chem. Int. Ed. Engl..

[48] Mohn F., Repp J., Gross L., Meyer G., Dyer M.S., Persson M. (2010). Reversible bond formation in a gold-atom-organic-molecule complex as a molecular switch. Phys. Rev. Lett..

[49] Pavliček N., Herranz-Lancho C., Fleury B., Neu M., Niedenführ J., Ruben M., Repp J. (2013). High-resolution scanning tunneling and atomic force microscopy of stereochemically resolved dibenzo[a,h]thianthrene molecules. Phys. Status Solidi B.

[50] Schuler B., Liu W., Tkatchenko A., Moll N., Meyer G., Mistry A., Fox D., Gross L. (2013). Adsorption geometry determination of single molecules by atomic force microscopy. Phys. Rev. Lett..

[51] Mistry A., Moreton B., Schuler B., Mohn F., Meyer G., Gross L., Williams A., Scott P., Costantini G., Fox D.J. (2015). The synthesis and STM/AFM imaging of “olympicene” benzo[cd]pyrenes. Chemistry.

[52] Mohn F., Gross L., Moll N., Meyer G. (2012). Imaging the charge distribution within a single molecule. Nat. Nanotechnol..

[53] Moll N., Schuler B., Kawai S., Xu F., Peng L., Orita A., Otera J., Curioni A., Neu M., Repp J. (2014). Image distortions of a partially fluorinated hydrocarbon molecule in atomic force microscopy with carbon monoxide terminated tips. Nano Lett..

[54] Schuler B., Collazos S., Gross L., Meyer G., Pérez D., Guitián E., Peña D. (2014). From perylene to a 22-ring aromatic hydrocarbon in one-pot. Angew. Chem..

[55] Sun Z., Boneschanscher M.P., Swart I., Vanmaekelbergh D., Liljeroth P. (2011). Quantitative atomic force microscopy with carbon monoxide terminated tips. Phys. Rev. Lett..

[56] Boneschanscher M.P., Ha S.K., Liljeroth P., Swart I. (2014). Sample corrugation affects the apparent bond lengths in atomic force microscopy. ACS Nano.

[57] Neu M., Moll N., Gross L., Meyer G., Giessibl F.J., Repp J. (2014). Image correction for atomic force microscopy images with functionalized tips. Phys. Rev. B.

[58] Weiss C., Wagner C., Temirov R., Tautz F.S. (2010). Direct imaging of intermolecular bonds in scanning tunneling microscopy. J. Am. Chem. Soc..

[59] Hapala P., Kichin G., Wagner C., Tautz F.S., Temirov R., Jelínek P. (2014). Mechanism of high-resolution STM/AFM imaging with functionalized tips. Phys. Rev. B.

[60] Hapala P., Temirov R., Tautz F.S. (2014). Origin of high-resolution IETS-STM images of organic molecules with functionalized tips. Phys. Rev. Lett..

[61] Zhang J., Chen P., Yuan B., Ji W., Cheng Z., Qiu X. (2013). Real-space identification of intermolecular bonding with atomic force microscopy. Science.

[62] Hämäläinen S.K., van der Heijden N., van der Lit J., den Hartog S., Liljeroth P., Swart I. (2014). Intermolecular contrast in atomic force microscopy images without intermolecular bonds. Phys. Rev. Lett..

[63] Kawai S., Sadeghi A., Xu F., Peng L., Orita A., Otera J., Goedecker S., Meyer E. (2015). Extended halogen bonding between fully fluorinated aromatic molecules. ACS Nano.

[64] PyProbe Web Interface. http://nanosurf.fzu.cz/ppr/.

[65] Jarvis S.P., Rashid M.A., Sweetman A.M., Leaf J., Taylor S., Dunn J.L., Moriarty P.J. (2015). Intermolecular artefacts observed in 2D C_60_ assemblies with NC-AFM.

[66] Theobald J.A., Oxtoby N.S., Phillips M.a., Champness N.R., Beton P.H. (2003). Controlling molecular deposition and layer structure with supramolecular surface assemblies. Nature.

[67] Korolkov V.V., Svatek S.A., Allen S., Roberts C.J., Tendler S.J.B., Taniguchi T., Watanabe K., Champness N.R., Beton P.H. (2014). Bimolecular porous supramolecular networks deposited from solution on layered materials: Graphite, boron nitride and molybdenum disulphide. Chem. Commun..

[68] Staniec P.A., Perdigão L.M.A., Saywell A., Champness N.R., Beton P.H. (2007). Hierarchical organisation on a two-dimensional supramolecular network. ChemPhysChem.

[69] Saywell A., Magnano G., Satterley C.J., Perdigão L.M.A., Champness N.R., Beton P.H., O’Shea J.N. (2008). Electrospray deposition of C_60_ on a hydrogen-bonded supramolecular network. J. Phys. Chem. C.

[70] Perdigão L.M.A., Perkins E.W., Ma J., Staniec P.A., Rogers B.L., Champness N.R., Beton P.H. (2006). Bimolecular networks and supramolecular traps on Au(111). J. Phys. Chem. B.

[71] Pawin G., Wong K.L., Kwon K.Y., Bartels L. (2006). A homomolecular porous network at a Cu(111) surface. Science.

[72] Mura M., Sun X., Silly F., Jonkman H.T., Briggs G.A.D., Castell M.R., Kantorovich L.N. (2010). Experimental and theoretical analysis of H-bonded supramolecular assemblies of PTCDA molecules. Phys. Rev. B.

[73] Silly F., Shaw A.Q., Castell M.R., Briggs G.A.D., Mura M., Martsinovich N., Kantorovich L. (2008). Melamine structures on the Au(111) surface. J. Phys. Chem. C.

[74] Staniec P.A., Perdigão L.M.A., Rogers B.L., Champness N.R., Beton P.H. (2007). Honeycomb networks and chiral superstructures formed by cyanuric acid and melamine on Au(111). J. Phys. Chem. C.

[75] Rahe P., Kittelmann M., Neff J.L., Nimmrich M., Reichling M., Maass P., Kühnle A. (2013). Tuning molecular self-assembly on bulk insulator surfaces by anchoring of the organic building blocks. Adv. Mater..

[76] Burke S.A., Topple J.M., Grütter P. (2009). Molecular dewetting on insulators. J. Phys. Condens. Matter.

[77] Burke S.A., Mativetsky J.M., Hoffmann R., Grütter P. (2005). Nucleation and submonolayer growth of C_60_ on KBr. Phys. Rev. Lett..

[78] Mativetsky J.M., Burke S.a., Hoffmann R., Sun Y., Grutter P. (2004). Molecular resolution imaging of C_60_ on Au(111) by non-contact atomic force microscopy. Nanotechnology.

[79] Mativetsky J.M., Burke S.A., Fostner S., Grutter P. (2007). Templated growth of 3,4,9,10-perylenetetracarboxylic dianhydride molecules on a nanostructured insulator. Nanotechnology.

[80] Pakarinen O.H., Mativetsky J.M., Gulans A., Puska M.J., Foster A.S., Grutter P. (2009). Role of van der Waals forces in the adsorption and diffusion of organic molecules on an insulating surface. Phys. Rev. B.

[81] Burke S.A., Ji W., Mativetsky J.M., Topple J.M., Fostner S., Gao H.J., Guo H., Grütter P. (2008). Strain induced dewetting of a molecular system: Bimodal growth of PTCDA on NaCl. Phys. Rev. Lett..

[82] Amrous A., Bocquet F., Nony L., Para F., Loppacher C., Lamare S., Palmino F., Cherioux F., Gao D.Z., Canova F.F. (2014). Molecular design and control over the morphology of self-assembled films on ionic substrates. Adv. Mater. Interfaces.

[83] Bocquet F., Nony L., Mannsfeld S.C.B., Oison V., Pawlak R., Porte L., Loppacher C. (2012). Inhomogeneous relaxation of a molecular layer on an insulator due to compressive stress. Phys. Rev. Lett..

[84] Pawlak R., Nony L., Bocquet F., Oison V., Sassi M., Debierre J.m., Loppacher C., Porte L. (2010). Supramolecular assemblies of 1,4-benzene diboronic acid on KCl(001). J. Phys. Chem..

[85] Rahe P., Nimmrich M., Kühnle A. (2012). Substrate templating upon self-assembly of hydrogen-bonded molecular networks on an insulating surface. Small.

[86] Kittelmann M., Rahe P., Nimmrich M., Hauke C.M., Gourdon A., Kühnle A. (2011). On-surface covalent linking of organic building blocks on a bulk insulator. ACS Nano.

[87] Rahe P., Nimmrich M., Greuling A., Schütte J., Stará I.G., Rybáček J., Huerta-Angeles G., Starý I., Rohlfing M., Kühnle A. (2010). Toward molecular nanowires self-assembled on an insulating substrate: Heptahelicene-2-carboxylic acid on Calcite (1014). J. Phys. Chem. C.

[88] Stepanow S., Lingenfelder M., Dmitriev A., Spillmann H., Delvigne E., Lin N., Deng X., Cai C., Barth J.V., Kern K. (2004). Steering molecular organization and host-guest interactions using two-dimensional nanoporous coordination systems. Nat. Mater..

[89] Schlickum U., Decker R., Klappenberger F., Zoppellaro G., Klyatskaya S., Ruben M., Silanes I., Arnau A., Kern K., Brune H. (2007). Metal-organic honeycomb nanomeshes with tunable cavity size. Nano Lett..

[90] Grill L., Dyer M., Lafferentz L., Persson M., Peters M.V., Hecht S. (2007). Nano-architectures by covalent assembly of molecular building blocks. Nat. Nanotechnol..

[91] Lafferentz L., Ample F., Yu H., Hecht S., Joachim C., Grill L. (2009). Conductance of a single conjugated polymer as a continuous function of its length. Science.

[92] Riss A., Wickenburg S., Gorman P., Tan L.Z., Tsai H.Z., de Oteyza D.G., Chen Y.C., Bradley A.J., Ugeda M.M., Etkin G. (2014). Local electronic and chemical structure of oligo-acetylene derivatives formed through radical cyclizations at a surface. Nano Lett..

[93] Albrecht F., Neu M., Quest C., Swart I., Repp J. (2013). Formation and characterization of a molecule-metal-molecule bridge in real space. J. Am. Chem. Soc..

[94] Elemans J.A.A.W., Lei S., de Feyter S. (2009). Molecular and supramolecular networks on surfaces: From two-dimensional crystal engineering to reactivity. Angew. Chem. Int. Ed. Engl..

[95] Slater (née Phillips) A.G., Beton P.H., Champness N.R. (2011). Two-dimensional supramolecular chemistry on surfaces. Chem. Sci..

[96] Kudernac T., Lei S., Elemans J.A.A.W., de Feyter S. (2009). Two-dimensional supramolecular self-assembly: Nanoporous networks on surfaces. Chem. Soc. Rev..

[97] Barth J.V., Costantini G., Kern K. (2005). Engineering atomic and molecular nanostructures at surfaces. Nature.

[98] Barth J.V. (2007). Molecular architectonic on metal surfaces. Annu. Rev. Phys. Chem..

[99] Frenking G., Frohlich N. (2000). The nature of the bonding in transition-metal compounds. Chem. Rev..

[100] Yurtsever A., Sugimoto Y., Tanaka H., Abe M., Morita S., Ondráček M., Pou P., Pérez R., Jelínek P. (2013). Force mapping on a partially H-covered Si(111)-(7×7) surface: Influence of tip and surface reactivity. Phys. Rev. B.

[101] Sharp P., Jarvis S., Woolley R., Sweetman A., Kantorovich L., Pakes C., Moriarty P. (2012). Identifying passivated dynamic force microscopy tips on H:Si(100). Appl. Phys. Lett..

[102] Jarvis S., Sweetman A., Bamidele J., Kantorovich L., Moriarty P. (2012). Role of orbital overlap in atomic manipulation. Phys. Rev. B.

[103] Lindner R., Kühnle A. (2015). On-surface reactions. ChemPhysChem.

